# Irisin prevents and restores bone loss and muscle atrophy in hind-limb suspended mice

**DOI:** 10.1038/s41598-017-02557-8

**Published:** 2017-06-06

**Authors:** Graziana Colaianni, Teresa Mongelli, Concetta Cuscito, Paolo Pignataro, Luciana Lippo, Giovanna Spiro, Angela Notarnicola, Ilenia Severi, Giovanni Passeri, Giorgio Mori, Giacomina Brunetti, Biagio Moretti, Umberto Tarantino, Silvia C. Colucci, Janne E. Reseland, Roberto Vettor, Saverio Cinti, Maria Grano

**Affiliations:** 1 Department of Basic Medical Science, Neuroscience and Sense Organs, University of Bari, 70124 Bari, Italy; 2 Department of Medicine-DIMED, Internal Medicine 3, University of Padova, 35128 Padova, Italy; 3 Department of Experimental and Clinical Medicine, Center of Obesity, United Hospitals, University of Ancona, 60020 Ancona, Italy; 4 Department of Clinical and Experimental Medicine, University of Parma, 43126 Parma, Italy; 5 Department of Clinical and Experimental Medicine, University of Foggia, 71100 Foggia, Italy; 6 Department of Orthopedics and Traumatology, Tor Vergata University of Rome, 00133 Rome, Italy; 7 Department of Biomaterials, Institute for Clinical Dentistry, University of Oslo, Blindern, N-0317 Oslo Norway; 8 Department of Emergency and Organ Transplantation, University of Bari, 70124 Bari, Italy

## Abstract

We previously showed that Irisin, a myokine released from skeletal muscle after physical exercise, plays a central role in the control of bone mass. Here we report that treatment with recombinant Irisin prevented bone loss in hind-limb suspended mice when administered during suspension (preventive protocol) and induced recovery of bone mass when mice were injected after bone loss due to a suspension period of 4 weeks (curative protocol). MicroCT analysis of femurs showed that r-Irisin preserved both cortical and trabecular bone mineral density, and prevented a dramatic decrease of the trabecular bone volume fraction. Moreover, r-Irisin protected against muscle mass decline in the hind-limb suspended mice, and maintained the fiber cross-sectional area. Notably, the decrease of myosin type II expression in unloaded mice was completely prevented by r-Irisin administration. Our data reveal for the first time that Irisin retrieves disuse‐induced bone loss and muscle atrophy. These findings may lead to development of an Irisin-based therapy for elderly immobile osteoporotic and physically disable patients, and might represent a countermeasure for astronauts subjected to microgravity-induced bone and muscle losses.

## Introduction

Disuse osteoporosis is a worldwide problem that affects patients suffering from poor walking and impaired physical ability up to immobility. Elderly, para- or hemiplegia after spinal cord injury or stroke^1, 2^ and vegetative states^3^ are examples of prolonged skeletal unloading that lead to decrease cortical mineral density and impairment of trabecular bone microarchitecture, resulting in skeletal fragility and increased fracture risk^4^. Data on fracture frequency in bedridden patients showed that 3.6% of 500 patients suffered from spontaneous fractures during a 6-year follow-up period^4^, suggesting that disuse osteoporosis is a clinically relevant issue for its related morbidity and mortality as well as for health-care costs.

Another well-documented unloading condition affecting the skeleton is the long exposure to microgravity during spaceflight. Astronauts are prone to bone loss at a rate of 0.5% to 1.5% per month^5^, equally to the loss found in postmenopausal women in 1 year^6, 7^. Even the return to Earth cannot fully restore bone loss of astronauts, who partially recover bone mineral density (BMD) in 1 year after returning from 4 to 6 months on the International space station^8^. As a result of weight-bearing removal, pathological changes affecting the skeleton are further exacerbated by concomitant onset of muscle atrophy. Indeed, astronauts experienced 4% to 8% muscle volume decline after the first week of shuttle mission^9^ and up to 10% reduction in strength of knee extensor muscles and 8% decrease in muscle fiber cross-sectional area (CSA) has been found after 17 days in microgravity environment^10^. Thus, the influence of microgravity has been studied in order to investigate the myosin phenotype changes in skeletal muscle fibers. Biopsies of *vastus lateralis* obtained from space shuttle astronauts were analyzed before and after 5- and 11-day spaceflights. According to this report^11^, fiber CSA of slow-twitch (Type I) and fast-twitch (Type II) fibers in post-flight biopsies were, respectively, 15% and 22% less than in preflight biopsies.

As during aging and immobility, these physiological changes caused by skeletal unloading determines onset of sarcopenia, which is characterized by a decrease in muscle fiber size (atrophy) and number (hypoplasia). Particularly, skeletal muscle in elderly subjects suffering from impaired walking showed greater atrophy of Type IIx fibers and a decrease in myosin heavy chains IIα and IIx mRNA levels^12^.

Several pharmacological and nutritional approaches have been tried to ameliorate the severe musculoskeletal injuries caused by mechanical unloading, but at present non-conclusive results have been obtained^13^. Since bone loss is often correlated with muscle wasting in several disuse-induced diseases, the interaction of these tissues has received increasing attention in recent years with mounting evidence suggesting the existence of a molecular crosstalk between muscle and bone. Among these molecules, the newly identified myokine Irisin is particularly relevant in the bone-muscle unit function, as we have recently shown^14–17^.

Irisin is a hormone-like molecule secreted from skeletal muscle in response to exercise both in mice and humans. Researchers originally identified Irisin as a myokine that targets white adipocytes to induce browning response and subsequently non-shivering thermogenesis^18^, but we demonstrated that Irisin also plays a central role in the control of bone mass, with positive effects on cortical mineral density and geometry *in vivo* ^15^. Recombinant Irisin (r-Irisin) induced increased cortical BMD, periosteal circumference and polar moment of inertia in long bones of healthy young mice^15^. Although Irisin clearly recapitulates some of the most important benefits of loading activity, such as physical exercise, its efficacy in unloading-induced osteoporosis has not been investigated yet. For this, we took advantage of the hind-limb suspended mouse, a murine model widely accepted for simulating weightlessness, in which load is prevented, but the passive muscular forces remain functional.

r-Irisin did not influence cancellous bone in healthy mice^15^, and as expected the trabecular microarchitecture remained intact. However, in the case of hind-limb suspended mice, the detrimental effect caused by the absence of load may affect the whole bone structure, with the major involvement of the trabecular compartment.

Here we show that r-Irisin treatment ameliorates disuse-induced osteoporosis and muscle atrophy in hind-limb suspended mice. We found that r-Irisin treatment markedly acted on cortical BMD with prevention of bone loss when hind-limb suspended mice were treated during suspension. Likewise, cortical BMD was completely recovered by r-Irisin injection in unloaded mice after a suspension period during which they developed expected bone loss. Furthermore, loss of trabecular BMD and bone volume fraction (BV/TV) in unloaded mice were also prevented by r-Irisin therapy. Moreover, we demonstrated that r-Irisin has a significant effect on muscle mass, known to be suffering from atrophy during unloading. The dramatic decrease in myosin type II expression (MyHC II) in vastus lateralis of suspended mice was completely prevented by r-Irisin.

## Results

Data presented herein are based on two different protocols of treatment. The preventive protocol, where hind-limb suspended mice were treated with vehicle or r-Irisin (100 µg kg^−1^/weekly) during a 4-week suspension period. Results from these two groups of mice were compared to a control group of mice kept under normal loading and treated with vehicle. In the curative protocol, hind-limb suspended mice were suspended and left untreated for 4 weeks and then treated with vehicle or r-Irisin (100 µg kg^−1^ weekly) for the following 4 weeks of suspension. Results from these two groups of mice were also compared to two control groups of mice, both treated with vehicle: rest mice, which were kept under normal loading, and reloaded mice, which were hindlimb-unloaded for 4 weeks followed by 4 weeks of re-ambulation with normal cage activities. Results of preventive and curative protocols have not been compared to each other. They have a different experimental timing because the preventive protocol was performed to investigate if irisin could inhibit disuse-induced bone loss, whereas the curative protocol was performed to investigate if irisin could restore a pre-existing bone loss caused by disuse.

### Body weight measurement

Body weights were not significantly different after treatment by either of the protocols; neither the preventive protocol (Table 1), nor the curative protocol (Table 2).

**Table 1:** Mouse body weight (grams).

Time	Rest-veh-inj	Unload-veh-inj	Unload-Irisin-inj
Day 0	23.75 ± 0.91	24.66 ± 0.66	23.66 ± 0.65
Day 28	26.00 ± 0.53^§^	25.77 ± 0.52	24.66 ± 0.66

Preventive Protocol: comparisons of Body Weights (grams) of male C57BL/6 mice in the three Experimental Groups before (day 0) and after hind-limb suspension (Day 28). Data are presented as mean ± SEM. ^§^p < 0.05 (p value Day 28 Vs Day 0). No significant differences were detected between groups of mice at the end of treatment.

**Table 2:** Mouse body weight (grams).

Time	Rest veh-inj	Unload veh-inj	Unload Irisin-inj	Reload veh-inj
Day 0	25.50 ± 0.95	25.00 ± 0.55	24.57 ± 0.36	23.33 ± 0.66
Day 56	28.50 ± 0.95^§§^	27.66 ± 0.27^§^	26.28 ± 1.01^§^	27.33 ± 1.15^§§^

Curative Protocol: comparisons of Body Weights (grams) of male C57BL/6 mice in the four Experimental Groups before (day 0) and after hind-limb suspension or reload activity (Day 56). Data are presented as mean ± SEM. ^§^p < 0.05, ^§§^p < 0.01 (p value Day 56 Vs Day 0). No significant differences were detected between groups of mice at the end of treatment.

### r-Irisin prevents disuse-induced bone loss

We have previously shown that r-Irisin, injected in healthy mice, stimulates cortical bone formation and recapitulates some of the most important benefits of physical exercise on the skeleton^15^. This suggested that Irisin might be a potential candidate therapy for preventing bone loss caused by absence of mechanical loading. We therefore injected hind-limb suspended mice with r-Irisin (100 µg kg^−1^) or vehicle once a week for 4 weeks (preventive protocol) and compared the effect to littermate mice kept in resting condition and injected with vehicle. As internal control, we also injected mice kept in resting condition with r-Irisin (100 µg kg^−1^).

**Figure 1:**
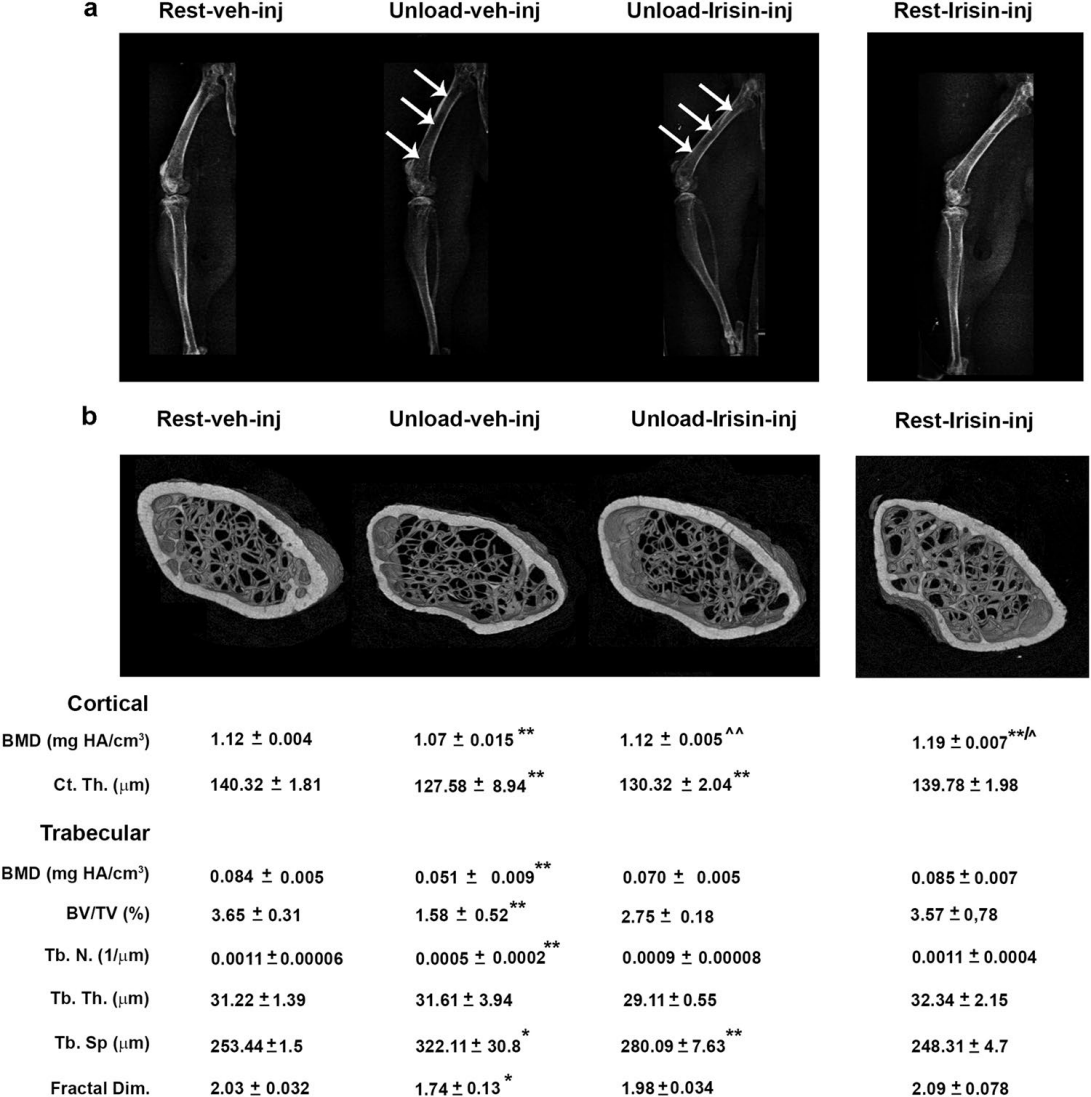
Treatment with r-Irisin prevents bone loss in femurs from hindlimb-suspended mice. (**a**) Contact radiographs of selected long bones from normal loading mice (Rest veh-inj) and unloaded mice treated with vehicle or recombinant Irisin (r-Irisin, 100 µg kg^−1^ *per* week for 28 days, mice were sacrificed 24 hours after last dose). Arrows indicate difference in radiodensity between femur of unloaded mice treated with vehicle and femur of unloaded mice treated with recombinant Irisin. (**b**) Representative micro-CT-generated section images and calculated cortical and trabecular parameters of femurs harvested from Rest mice vehicle- or Irisin-injected and Unload mice vehicle- or r-Irisin-injected. Cortical bone parameters included bone mineral density (BMD) and cortical thickness (Ct.Th). Trabecular bone parameters included bone mineral density (BMD), bone volume/total volume (BV/TV), trabecular number (Tb.N), trabecular thickness (Tb.Th), trabecular separation (Tb Sp) and Fractal Dimension. Data are presented as mean ± SEM. n = 7–8 mice per group. All data were normally distributed according to the Shapiro-Wilk normality test and analyzed by one-way ANOVA and Bonferroni’s post hoc analysis. Cohen’s d values were measured for non-significant differences of results and can be found as Supplementary Table S1. *p ≤ 0.05, **p ≤ 0.01 versus Rest vehicle-injected mice. ^^^ *p* ≤ 0.05, ^^^^ *p* ≤ 0.01 versus Unload vehicle-injected mice.

X-ray imaging of intact animals showed a generalized increase in radio density in the long bones of unloaded mice treated with r-Irisin compared to those treated with vehicle (Fig. 1a). Through visual inspection of radiographs, we observed in the femurs the major difference in radio density among groups of mice, as indicated by arrows. Moreover, as previously demonstrated^15^, X-ray imaging showed increased radio density in both femora and tibia of Rest irisin-treated mice compared with Rest mice treated with vehicle (Fig. 1a). Qualitative observations of microCT generated section images of femurs (Fig. 1b) showed a severe decrease of bone mass at both cortical and trabecular sites in unload mice treated with vehicle, but no marked difference was observed between unloaded mice treated with Irisin and control mice kept in resting condition and injected with vehicle (rest-veh-inj) (Fig. 1b). By microCT analysis, we found that cortical BMD in femurs from unloaded mice injected with vehicle was ~4% (p < 0.01) lower than cortical BMD of control mice (Fig. 1b). Interestingly, the unloaded mice injected with r-Irisin had no loss of cortical BMD (p = 0.69) compared to control mice, thus indicating that unloading-induced deterioration of the mineral component at cortical site was completely prevented by r-Irisin administration. As expected, cortical BMD was ~6.25% higher in Rest mice treated with r-irisin (p < 0.01) compared with vehicle controls (Fig. 1b). In contrast, cortical thickness (Ct.Th.) that was a significant ~9% lower than in control mice, remained unaffected by r-Irisin treatment in both unloaded and rest groups.

Trabecular BMD was dramatically reduced by ~39% (p < 0.01) in unloaded mice compared to control mice, whereas r-Irisin treatment protected from this BMD decline (p = 0.10). Furthermore, the unloading condition dramatically reduced BV/TV with respect to control mice, whereas in unloaded mice treated with r-Irisin this effect was less dramatic, resulting in an attenuated decline in BV/TV by ~32% and in trabecular number (Tb.N) by ~37%. Reliably, the increase of trabecular separation (Tb.Sp) observed in vehicle-treated unloaded mice (~27%; p < 0.05) was attenuated by ~17% in mice treated with r-Irisin. Instead, trabecular thickness (Tb.Th) was not affected by unloading and no significant change were observed in unloaded mice, treated either with vehicle or with r-Irisin, compared to control mice. Of note, the fractal dimension, an index of optimal micro-architectural complexity of trabecular bone, which decreased by ~14% (p < 0.05) in unloaded mice injected with vehicle versus control mice, was preserved by r-Irisin (p = 0.35) (Fig. 1b).

As we already showed for tibia^15^, r-irisin did not induce change in microstructural parameters of trabecular bone at the distal femurs: BMD, BV/TV, Tb.N, Tb.Th, and Tb.Sp were unchanged in r-irisin-treated Rest mice compared with vehicle controls (Fig. 1b).

### r-Irisin inhibits sclerostin increase and osteoprotegerin decrease caused by unloading *in vivo* and restores osteoblastogenesis in *ex vivo* cultures from unloaded mice

**Figure 2:**
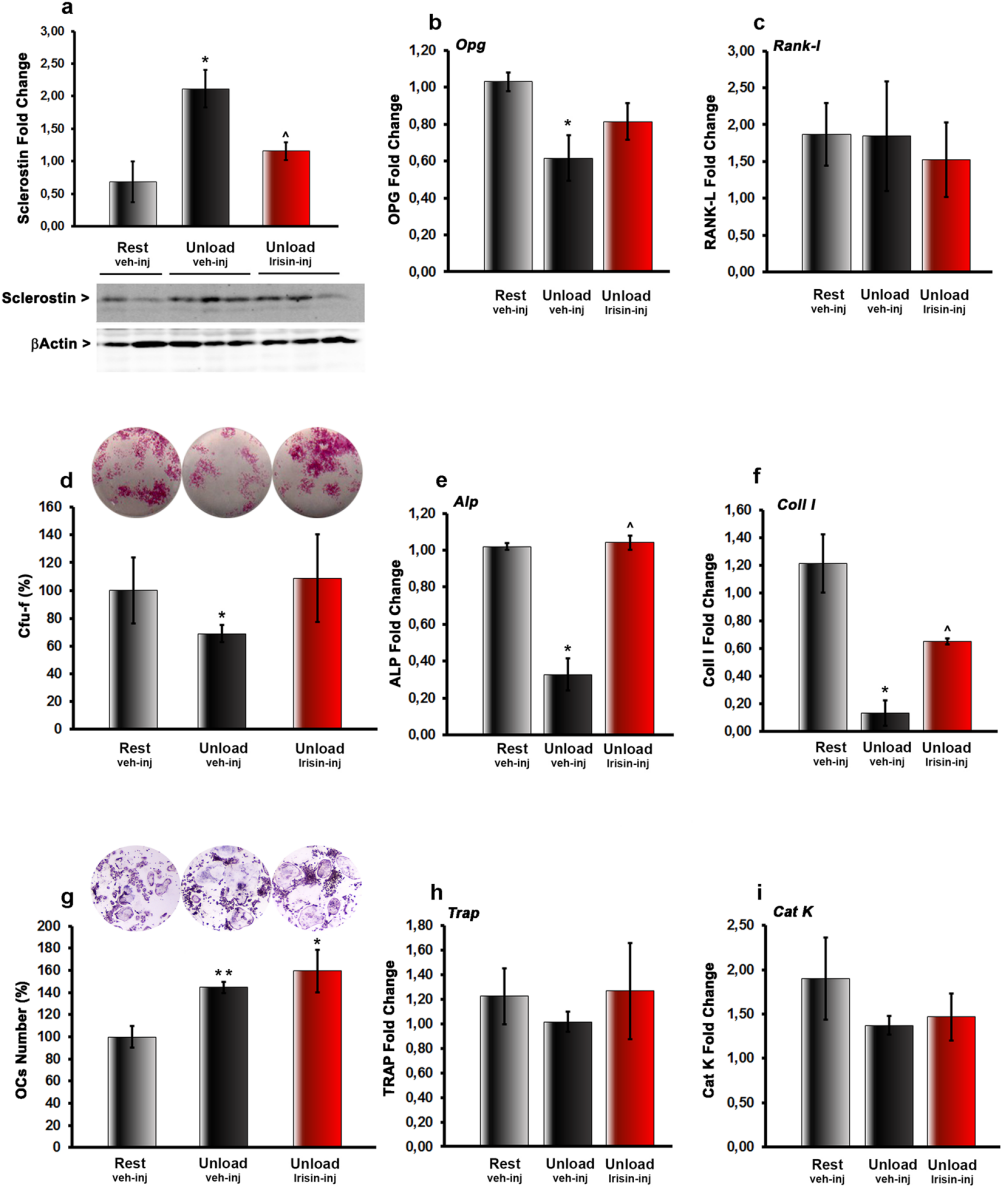
Treatment with r-Irisin inhibits sclerostin increase and Opg decrease caused by unloading *in vivo*. (**a**) Western immunoblotting and densitometric quantitation of sclerostin expression versus control loading (β-actin) in long bones (depleted of bone marrow) harvested from Rest vehicle-injected and Unload vehicle- or r-Irisin-injected mice (r-Irisin, 100 µg kg^−1^ *per* week for 28 days, mice sacrificed 24 hours after last dose). (**b**) *Opg* and (**c**) *Rank-l* mRNA expression (qPCR) in long bones (depleted of bone marrow) harvested from Rest vehicle-injected and Unload vehicle- or r-Irisin-injected mice. (**d**) *Ex vivo* Cfu-f formation (%), (**e**) *Alp* and (**f**) *Collagen I* mRNA expression (qPCR) in *ex vivo* cultures obtained from bone marrow harvested from Rest vehicle-injected and Unload vehicle- or r-Irisin-injected mice. (**g**) TRAP-positive osteoclast formation (%), (**h**) *Trap* and (**I**) *Cathepsin K* mRNA expression (qPCR) in *ex vivo* cultures obtained from bone marrow harvested from Rest veh-injected and Unload vehicle- or r-Irisin-injected mice. Data are presented as mean ± SEM. n = 3 mice per group. All data were normally distributed according to the Shapiro-Wilk normality test and analyzed by one-way ANOVA and Bonferroni’s post hoc analysis. *p ≤ 0.05, **p ≤ 0.01 versus Rest vehicle-injected mice. ^^^ *p* ≤ 0.05 versus Unload vehicle-injected mice.

As expected, after 4 weeks of unloading, sclerostin expression in long bones was higher than in control mice (~210%, p < 0.05) (Fig. 2a), whereas no significant difference was detected in r-Irisin treated mice, although it tended to be higher than control. Unloading decreased the gene expression of *Opg* with respect to control mice (p < 0.05) (Fig. 2b) without affecting the *Rankl* mRNA level (Fig. 2c). Nevertheless, r-Irisin treatment attenuated the *Opg* decrease, with no effect on *Rankl* expression, thus resulting in a *Rank-l*/*Opg* ratio similar to mice kept under normal loading.

*Ex vivo* primary cell culture experiments were performed to investigate if the effect of irisin was persistent after treatment *in vivo*. *Ex-vivo* cultures of bone marrow stromal cells harvested from unloaded mice showed a significant reduction in colony forming-fibroblastoid (Cfu-f) at day 10 (Fig. 2d). This pronounced decrease in osteoblast differentiation was demonstrable further by the reduced expression of Alkaline Phosphatase (*Alp*) and Collagen I (*Coll I*) mRNA (Fig. 2e,f). Conversely, *ex vivo* cultures of bone marrow stromal cells derived from unloaded mice treated with r-Irisin formed Cfu-f colonies similarly to control mice (Fig. 2d). Moreover, r-Irisin treatment inhibited the decrease in *Alp* and *Coll I* mRNA expression (Fig. 2e,f). We next investigated the effects of r-Irisin treatment on *ex-vivo* osteoclastogenesis. Bone marrow cells from unloaded mice injected with vehicle, cultured in the presence of Rankl, showed a significant increase in osteoclast (OC) number compared with cells from control mice (Fig. 2g). We observed that OC formation was higher than control also in bone marrow cells from unloaded mice treated with r-Irisin, even though, in both groups of hind-limb suspended mice, this was accompanied by unchanged expression of Phosphatase Acid Tartrate Resistant (*Trap*) and Cathepsin K (*Cat K*) mRNA (Fig. 2h,i).

### r-Irisin prevents disuse-induced muscle atrophy

**Figure 3:**
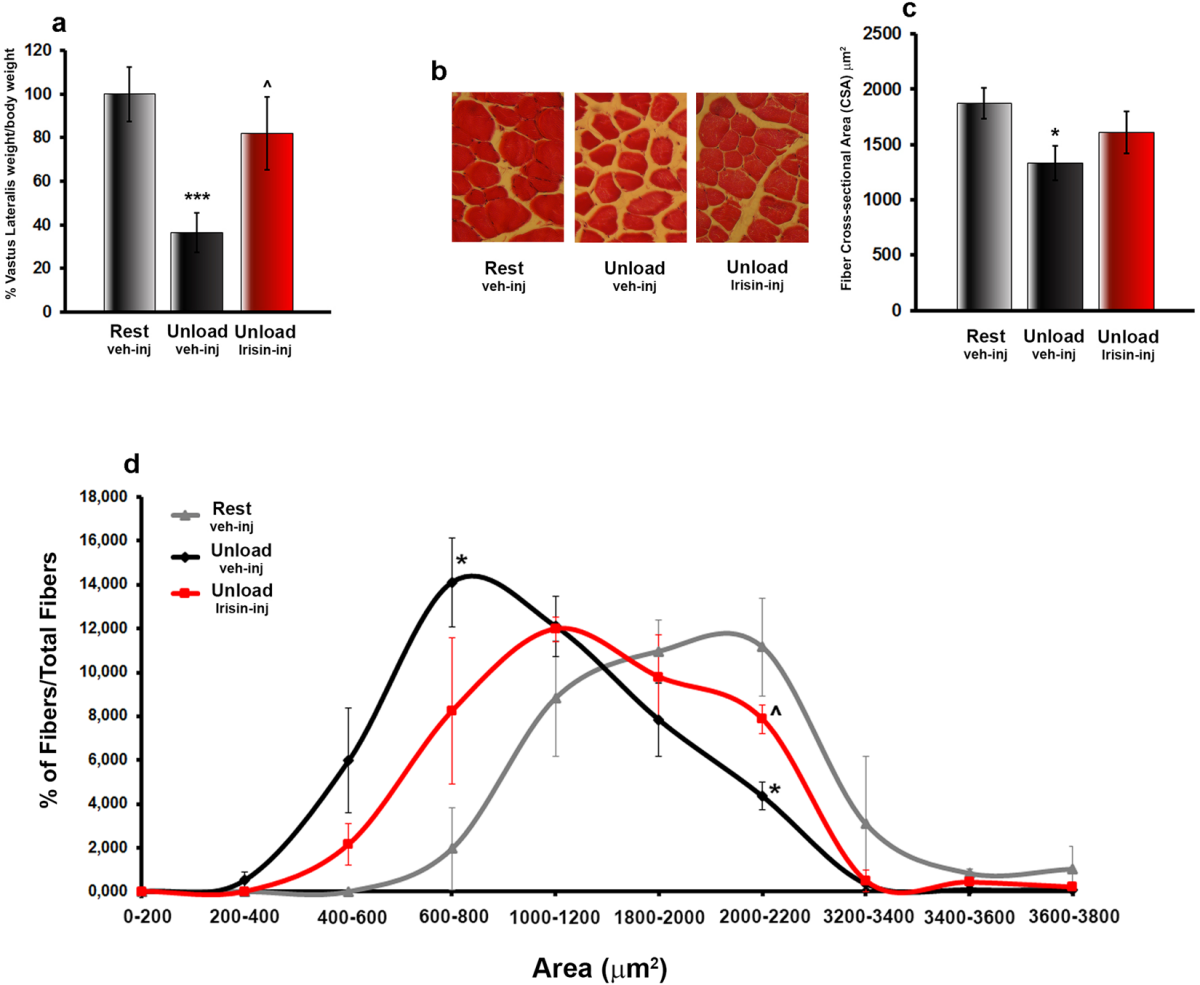
Treatment with r-Irisin prevents muscle wasting in hindlimb-suspended mice. (**a**) Vastus lateralis weight normalized to total body weight from normal loading mice (Rest veh-inj) and unloaded mice treated with vehicle or recombinant Irisin (r-Irisin, 100 µg kg^−1^ *per* week for 28 days, mice sacrificed 24 hours after last dose). (**b**) Photomicrographs of hematoxylin and eosin stained sections of vastus lateralis from Rest vehicle-injected and Unload vehicle- or Irisin-injected mice (magnification: 20x). (**c**) Quantitative assessments of Cross-Sectional Area (CSA) and (**d**) CSA area distribution of fibers from vastus lateralis harvested from normal loading mice (Rest vehicle-inj) and unloaded mice treated with vehicle or r-Irisin. Data are presented as mean ± SEM. n = 6–7 mice per group. All data were normally distributed according to the Shapiro-Wilk normality test and analyzed by one-way ANOVA and Bonferroni’s post hoc analysis. *p ≤ 0.05, ***p ≤ 0.001 versus Rest vehicle-injected mice. ^^^ *p* ≤ 0.05 versus Unload vehicle-injected mice.

It has been demonstrated that mice treated with r-Irisin displayed a higher number of FNDC5 positive muscle fibers compared to mice injected with vehicle, indicating that Irisin synthesis may be enhanced by its autocrine action^15^. In order to determine whether the muscle atrophy, typical of mechanical unloading, is prevented by r-Irisin treatment, we analysed *vastus lateralis* harvested from mice treated accordingly to the preventive protocol. Unloading resulted in a ~63% reduction in weight of *vastus lateralis*/body weight (p < 0.001) (Fig. 3a), whereas treatment with r-irisin induced no difference from resting control mice (p = 0.39).

In order to determine whether the reduction in muscle weight could be attributable to a reduction in overall muscle fiber CSA, hematoxylin and eosin (H&E) staining was performed on *vastus lateralis* sections (Fig. 3b). Measurements of CSA showed that fibers from unloaded mice treated with vehicle had lower area than control mice (p < 0.05), whereas fibers from unloaded mice treated with r-Irisin had similar size as those of control mice (p = 0.44) (Fig. 3c). Moreover, consistently with an overall reduction in fiber sizes, the frequency distribution of muscle fiber CSA was shifted leftward in the unloaded group treated with vehicle. Expectedly, unloaded mice treated with r-Irisin displayed fiber area distribution similarly to control mice (Fig. 3d).

**Figure 4:**
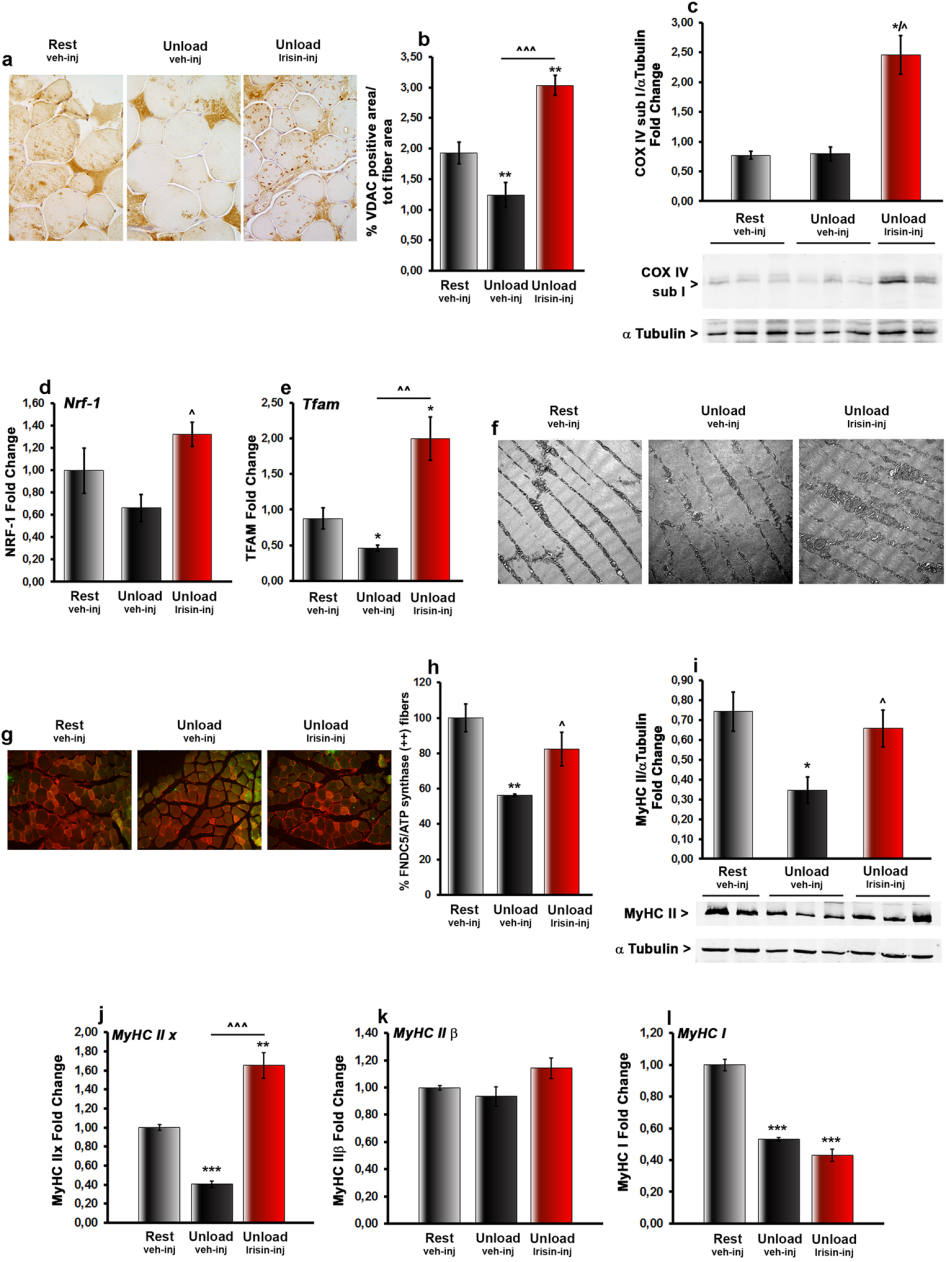
Treatment with r-Irisin inhibits myosin heavy chain type II decrease caused by unloading *in vivo*. (**a**) Representative images of immunohistochemistry staining of VDAC protein in *vastus lateralis* from normal loading mice (Rest vehicle-inj) and unloaded mice treated with vehicle or recombinant Irisin (r-Irisin, 100 µg kg^−1^ *per* week for 28 days, mice sacrificed 24 hours after last dose) (magnification: 40x). (**b**) Quantitative assessment of percentage of VDAC staining. (**c**) Densitometric quantitation of cytomchrome c oxidase subunit I (COX IV) expression versus control loading (α-tubulin) in vastus lateralis isolated from Rest vehicle-injected and Unload vehicle- or r-Irisin-injected mice. (**d**) *Nrf-1* and (**e**) *Tfam* mRNA expression (qPCR) in vastus lateralis harvested from Rest vehicle-injected and Unload vehicle- or Irisin-injected mice. (**f**) Electron microscope images. (**g**) Fluorescent micrographs of vastus lateralis sections of Rest vehicle-injected and Unload vehicle- or r-Irisin-injected mice immunolabeled for FNDC5 (green) and ATP synthase (red) (magnification: 20x). (**h**) Quantitative assessment of percentage of co-localization of FNDC5 and ATP synthase positive fibers (**i**) Densitometric quantitation of myosin heavy chain type II (MyHC II) expression versus control loading (α-tubulin) in vastus lateralis isolated from Rest vehicle-injected and Unload vehicle- or r-Irisin-injected mice. (**j**) *MyHC IIx*, (**k**) *MyHC II*β and (**l**) *MyHC I* mRNA expression (qPCR) in *vastus lateralis* harvested from Rest vehicle-injected and Unload vehicle- or Irisin-injected mice. Data are presented as mean ± SEM. n = 3–4 mice per group. All data were normally distributed according to the Shapiro-Wilk normality test. Results from Fig. 4 were analyzed by one-way ANOVA and Bonferroni’s post hoc analysis. *p ≤ 0.05, **p ≤ 0.01, ***p ≤ 0.001 versus Rest vehicle-injected mice. ^^^ *p* ≤ 0.05, ^^^^^ *p* ≤ 0.001 versus Unload vehicle-injected mice.

Since several forms of muscle wasting can affects mitochondria, their morphology was monitored. Immunohistochemistry revealed that muscle sections of unloaded mice had less abundant mitochondrial voltage-dependent anion channel (VDAC) staining, used as an index of mitochondrial content, whereas muscle from r-Irisin treated animals showed VDAC-positivity similar to control mice (Fig. 4a). Morphometry of immunoreactive areas fully confirmed the visual evidence (Fig. 4b). To further analyse the functional capability of newformed mitochondria, we measured cytomchrome c oxidase subunit I (COX IV) protein levels, an enzyme of the respiratory electron transport chain. Although COX IV expression was unchanged in unloaded mice with respect to control mice, its levels were significantly elevated by 2.5-fold in *vastus lateralis* of unloaded mice treated with r-Irisin compared with controls (Fig. 4c).

Furthermore, r-Irisin increased the gene expression of nuclear respiratory factor 1 (NRF1) and mitochondrial transcription factor A (TFAM) in unloaded mice (Fig. 4d,e). In line with these data electron microscopy showed marked decrease in mitochondrial density in vastus lateralis fibers from unloaded mice. Nevertheless, atrophic muscle fibers also showed a disorganized mitochondrial network in comparison to muscle fibers of control mice which displayed the typical striated distribution of intermyofibrillar mitochondria (Fig. 4f). Increased mitochondrial biogenesis (mitochondrial density and size), was observed in muscle fibers from unloaded mice injected with r-Irisin, even when they were compared to control mice (Fig. 4f). Altogether these data support a stimulated mitochondrial biogenesis, in line with recent data obtained *in vitro* ^19^. Additionally, immunofluorescence revealed that co-localization of FNDC5 positive and ATPsynthase positive fibers was reduced in unloading condition with respect to control mice (Fig. 4g), as also confirmed by quantification of percentage of FNDC5/ATPsynthase fibers (Fig. 4h). The treatment with r-Irisin preserved the number of fibers co-expressing FNDC5 and ATPsynthase, thus indicating a possible correlation between the autocrine-induced FNDC5/Irisin expression pattern and the mitochondrial content in skeletal muscle (Fig. 4g,h).

Furthermore, western blotting analysis demonstrated a dramatic reduction in myosin type II (MyHC II) expression in *vastus lateralis* from unloaded mice treated with vehicle compared to control mice (p < 0.05) (Fig. 4i). This significant twofold decrease of MyHC II expression was abolished when unloaded mice were treated with r-Irisin, demonstrating that MyHC II is sensible to Irisin action. To further investigate which sub-type of MyHC II was particularly involved in Irisin-dependent prevention of unloading-induced muscle wasting, we explored the expression of myosin type IIα (MyHC IIα), type IIx (MyHC IIx) and type IIβ (MyHC IIβ), which are classified in this sequence from the slowest to the fastest isoform of myosin type II. Quantitative PCR showed that *MyHC IIx* mRNA was strongly downregulated in muscle from unloaded mice treated with vehicle versus control mice (p < 0.001). r-Irisin treatment, not only inhibited its decline, but also increased its expression with respect to control mice (p < 0.01) (Fig. 4j). *MyHC IIb* mRNA expression was unchanged in unloaded mice, either treated with vehicle or with r-Irisin, respect to control mice (Fig. 4k). No detectable levels of MyHC IIα were observed in either of the groups of mice (data not shown). Moreover, quantitative PCR showed that *MyHC I* mRNA expression was lower than control in both vehicle- and r-Irisin treated unloaded mice (Fig. 4l).

### r-Irisin did not influence the regenerative potential of muscle stem cells

**Figure 5:**
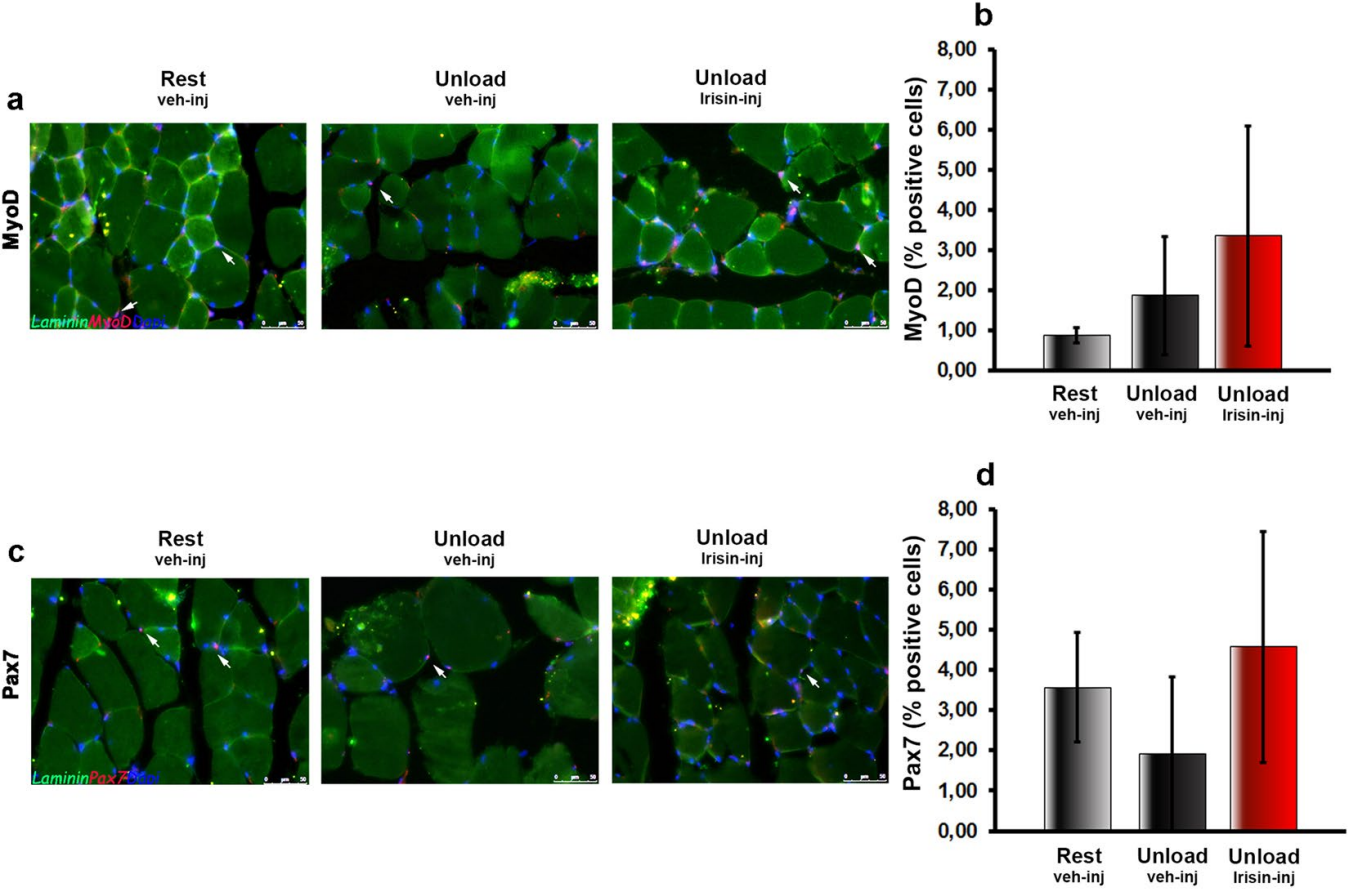
Treatment with r-Irisin does not affect satellite cells in hindlimb-suspended mice. Fluorescent micrographs of *vastus lateralis* sections from Rest vehicle-injected and Unload vehicle- or Irisin-injected mice, immunostained (red) for MyoD (**a**) and Pax7 (**c**) and counterstained for Laminin (green) and DAPI (blue). The percentage of MyoD positive (**b**) and Pax7 positive (**d**) cells are quantified. Data are presented as mean ± SEM. n = 3 mice per group. All data were normally distributed according to the Shapiro-Wilk normality test and analyzed by one-way ANOVA and Bonferroni’s post hoc analysis.

Since we have shown that Irisin treatment was able to prevent the damage of unloaded skeletal muscle *in vivo*, we aimed to evaluate if muscle satellite stem cells proliferation and differentiation, the key steps in the regeneration of skeletal muscle, were involved in this process. We did not observe any significant difference in the percentage of MyoD+/DAPI+ satellite cells in *vastus lateralis* (Fig. 5a, white arrows) in the three groups of mice (Fig. 5b). Likewise, no differences were observed on Pax7-positive cells after r-Irisin treatment (Fig. 5c,d). These results suggest that the effect of Irisin on muscle atrophy does not involve satellite cells.

### r-Irisin restores disuse-induced bone loss

In order to investigate if Irisin was effective in retrieving an already developed bone loss, we left untreated hind-limb unloaded mice for 4 weeks and then we treated them with r-Irisin (100 µg kg^−1^) or vehicle once a week for a 4-week period during which mice were left suspended. In addition to the control group of mice kept in resting condition throughout the experiment (8 weeks), we also used a group of reload mice, as internal control of loading, which were hindlimb-unloaded for 4 weeks followed by 4 weeks of re-ambulation that involved normal cage activities.

**Figure 6:**
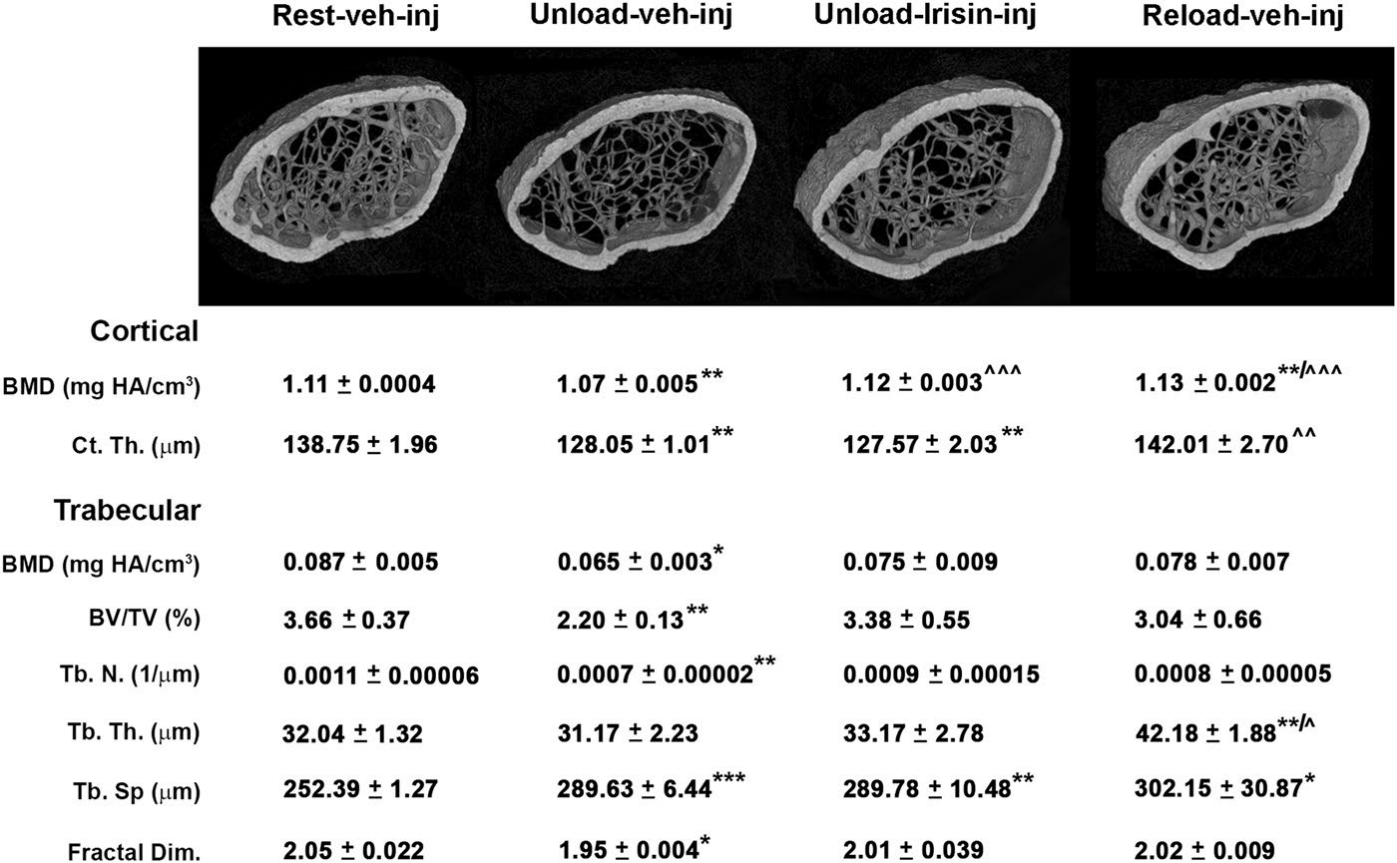
Treatment with r-Irisin recovers bone loss in femurs from hindlimb-suspended mice. Representative microCT-generated section images and calculated cortical and trabecular parameters of femurs obtained from normal loading mice (Rest vehicle-inj), unloaded mice treated with vehicle or recombinant Irisin (first r-Irisin injection after 4 weeks of hindlimb suspension, at the dose of 100 µg kg^−1^ *per* week for 28 days) and reloaded mice (Reload veh-inj). Cortical bone parameters included bone mineral density (BMD) and cortical thickness (Ct.Th). Trabecular bone parameters included bone mineral density (BMD), bone volume/total volume (BV/TV), trabecular number (Tb.N), trabecular thickness (Tb.Th), trabecular separation (Tb Sp) and Fractal Dimension. Data are presented as mean ± SEM. n = 6–7 mice per group. All data were normally distributed according to the Shapiro-Wilk normality test and analyzed by one-way ANOVA and Bonferroni’s post hoc analysis. Cohen’s d values were measured for non-significant differences of results and can be found as Supplementary Table S1. *p ≤ 0.05, **p ≤ 0.01, ***p ≤ 0.001 versus Rest vehicle-injected mice. ^^^ *p* ≤ 0.05, ^^^^ *p* ≤ 0.01, ^^^^^ *p* ≤ 0.001 versus Unload vehicle-injected mice.

MicroCT generated section images (Fig. 6) showed marked damage at both cortical and trabecular sites of femurs from unloaded mice treated with vehicle. Qualitative observations of femurs were confirmed by microCT analysis of bone parameters. Cortical BMD was decreased in femurs from unloaded mice compared to control mice (Fig. 6), whereas treatment with r-Irisin recovered cortical BMD to that of control mice (p = 0.19). Similarly, it was also fully recovered by reloading activity, suggesting that the effect of r-Irisin on cortical BMD is likely reloading-mimetic. In contrast, both unloaded mice, either treated with vehicle or with r-Irisin, showed ~7.71% (p < 0.01) and ~8.06% (p < 0.01) decrease of Ct.Th. versus control mice, respectively. Conversely, reloaded mice fully retrieve femoral Ct. Th to control level (p = 0.35) (Fig. 6).

Like cortical, also trabecular BMD was markedly reduced in unloaded mice treated with vehicle with respect to control mice (p < 0.05), whereas trabecular BMD in unloaded mice treated with r-Irisin was not significantly different from control mice (p = 0.24), Likewise, trabecular BMD in reloaded mice was not significantly different from control mice (p = 0.38), but not fully recovered (Fig. 6). As expected, the unloading condition strongly decreased BV/TV, whereas treatment with r-Irisin retrieved loss of trabecular mass, resulting in a ~32% of the decline in BV/TV similar to rescue by reloading activity. The effect of r-Irisin was exerted on trabecular number (Tb.N), which was significantly reduced in unloaded mice treated with vehicle (p < 0.01), whereas treatment with r-Irisin recovered this by ~17%, still resulting in a not significant reduction (p = 0.32) in Tb.N of ~12.98% compared to control mice. Interestingly, also in reloaded mice, Tb.N was partially recovered by ~8.61%, but to a lower extend than unloaded mice treated with r-Irisin. Consistently with the decreased number of trabeculae in femurs of vehicle-treated unloaded mice, trabecular separation (Tb.Sp) was ~14.75% higher than in control mice (p < 0.001). Surprisingly, Tb.Sp was not reduced in unloaded mice treated with r-Irisin and remained higher than in control mice (p < 0.01), as well as in reloaded mice (p < 0.05). Moreover, trabecular thickness (Tb.Th) was not affected by unloading conditions, however showed a tendency, even not significant, to increase by ~3.51% in unloaded mice treated with r-Irisin compared to control mice. Interestingly, reloaded mice also had a marked increase of Tb.Th (p < 0.01) compared to control mice, thus suggesting that the effect of r-Irisin on Tb.Th may mimic that of reloading activity. Furthermore, fractal dimension, which was reduced in unloaded mice compared to control mice (p < 0.05), returned to control level in unloaded mice treated with r-Irisin (p = 0.37) and, similarly, in reloaded mice (p = 0.44) (Fig. 6).

**Figure 7:**
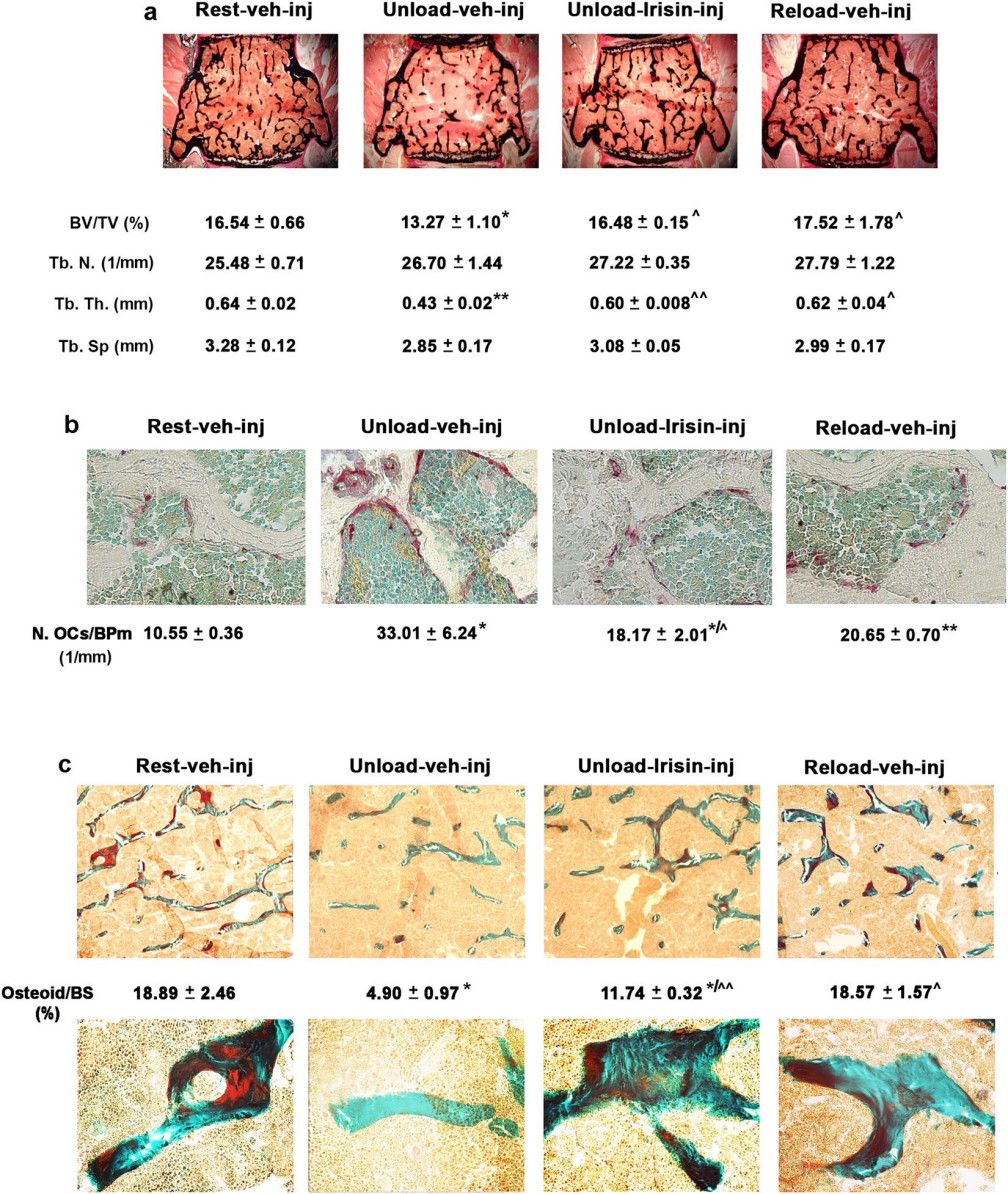
Treatment with r-Irisin recovers bone loss in vertebrae from hindlimb-suspended mice. (**a**) Von Kossa-stained vertebral sections and trabecular bone parameters from normal loading mice (Rest vehicle-inj), unloaded mice treated with vehicle or recombinant Irisin (first r-Irisin injection after 4 weeks of hindlimb suspension, at the dose of 100 µg kg^−1^ *per* week for 28 days) and reloaded mice (Reload vehicle-inj) (magnification: 2.5x). (**b**) Representative images of tartrate-resistant acid phosphatase-stained osteoclasts in vertebral sections, together with osteoclast counts per bone perimeter (BPm). (magnification: 40x). (**c**) Representative images of Goldner’s Masson Trichrome-stained vertebral sections (magnification: 10x) and photomicrograph of details taken at 40x, together with measurement of percentage of osteoid per bone surface (BS). n = 4–5 mice per group. Data are presented as mean ± SEM. All data were normally distributed according to the Shapiro-Wilk normality test and analyzed by one-way ANOVA and Bonferroni’s post hoc analysis. *p ≤ 0.05, **p ≤ 0.01 versus Rest vehicle-injected mice. ^^^ *p* ≤ 0.05, ^^^^ *p* ≤ 0.01 versus Unload vehicle-injected mice.

We also investigated whether r-Irisin was able to retrieve bone loss in vertebral bodies during unloading (Fig. 7a). By analyzing L3-L4 vertebra, we found that BV/TV in unloaded mice treated with vehicle was dramatically reduced by ~19.78% versus control mice (p < 0.05), whereas unloaded mice treated with r-Irisin displayed a fully recovered trabecular bone mass (p = 0.94). Likewise, reloaded mice had BV/TV similar to control mice (p = 0.58). The effect of r-Irisin on vertebral bone mass was mainly exerted on the thickness of trabeculae. In fact, the reduction of Tb.Th in unloaded mice treated with vehicle (p < 0.01 versus control mice), was rescued in mice treated with r-Irisin (p = 0.21 versus control mice), as well as in reloaded mice (p = 0.68 versus control mice) (Fig. 7a).

Knowing that absence of mechanical load induces bone loss primarily exacerbating bone resorption, we quantified the number of osteoclasts (OCs) on trabecular bone. As shown in Fig. 7b, in unloaded mice treated with vehicle, OCs number per bone perimeter (OCs/BPm) was ~212% higher than in control mice (p < 0.05), whereas r-Irisin strongly reduced the number of OCs, although it remained higher than control (p > 0.05). Consistently, also reloading activity decreased the number of OCs with respect to unloaded mice treated with vehicle, but did not fully restore OCs number back to control level (Fig. 7b).

We also observed a ~74% reduction in the area of osteoid surface to bone surface (OS/BS) in unloaded mice treated with vehicle compared to control mice (p < 0.05), whereas in unloaded mice treated with r-Irisin a strong trend toward increased OS/BS was observed, albeit it was still lower than in control mice (p > 0.05). Conversely, reloading activity fully recovered the osteoid surface area to that of control mice (Fig. 7c).

## Discussion

The aim of our study was to determine the effects of recombinant Irisin on the musculoskeletal system of mice exposed to hind-limb unloading. Our results clearly show that r-Irisin treatment during four weeks of suspension strikingly acts on both cortical and trabecular BMD, whose disuse-induced decrease was prevented. Likewise, cortical and trabecular BMD were also recovered when r-Irisin was administered to mice after 4 weeks of suspension, with developed bone loss. Moreover, at trabecular site, r-Irisin therapy was effective in preventing or restoring BV/TV decline in both protocols of suspension. Furthermore, we also show that r-Irisin prevented disuse-induced wasting of muscle mass, thus impeding the progression towards muscle atrophy.

The hind-limb suspension model is well accepted as ground-based analog to ascertain the effects of unloading conditions on the properties of the musculoskeletal system^20^. As humans forced to short- or long-term immobility, hind-limb suspended mice experience altered balance of muscle and bone metabolism, resulting in concomitant loss of both tissues^21, 22^. In accordance with previous findings^23–25^, our µCT analysis on femurs from unloaded mice, revealed notable deterioration of both cortical and trabecular bone microarchitecture.

For the preventive protocol, r-Irisin was administered during hind-limb unloading, starting from the beginning of suspension. This therapy was able to entirely prevent the decline of cortical BMD, whereas the loss of trabecular BMD, BV/TV and fractal dimension were partially prevented. Fractal dimension is an index of optimal micro-architectural complexity that indicates how irregular structures, such as trabeculae in the case of bone, tends to fill space. Specifically, under pathological conditions, trabecular shape and bone surfaces appear less regular and less complex than healthy bone^26^. Several studies reported that fractal dimension is lower in the osteoporotic population compared to healthy groups^27–29^ and it has been found positively correlated to BMD, indicating that evaluation of fractal dimension could be crucial for the diagnosis of osteoporosis^30^.

In healthy mice, r-Irisin did not influence cancellous bone of femurs, where the trabecular microarchitecture was already intact before treatment, as we already demonstrated for the tibia^15^. Conversely, the disuse condition affected the whole bone structure, with the major involvement of the trabecular compartment, which was instead protected by the action of Irisin.

In compliance with the curative protocol, to investigate whether Irisin was effective in recovering an already developed bone loss, r-Irisin was administered after four weeks of suspension and during the following four weeks of treatment mice were left suspended, Additionally, we also examined the efficacy of r-Irisin in comparison with reloading condition, in order to evaluate whether the Irisin-based therapy could mimic the action of re-ambulation. MicroCT analysis showed that loss of cortical and trabecular BMD, as well as BV/TV in unloaded mice were restored by r-Irisin therapy, as also observed in reloaded mice, clearly demonstrating a loading-mimetic activity of the myokine. However, despite most of similar actions observed between r-Irisin therapy and the re-ambulation, we found that cortical thickness was fully recovered only by reloading activity.

Despite a previous observation reporting that unloading has a greater effect on cortical compared to trabecular bone^21^, several studies have shown that the bone reaction to unloading also involves a strong decrease in trabecular bone mass^23–25^. In agreement with these latter results, we observed trabecular BV/TV decline in femurs of mice kept unloaded either for 4 or 8 weeks, according to the two different intervention protocols. Loss of bone mass in vertebrae, however, did not occur after 4 weeks of suspension (date not shown), but only when mice were unloaded for 8 weeks. Remarkably, treatment with r-Irisin fully restored the trabecular bone mass in vertebrae. These results demonstrate that, in the presence of impaired bone mass, the therapeutic potential of Irisin occurs equally on both bone compartments.

As we previously demonstrated, the expression of sclerostin, one of the Wnt/β-catenin pathway inhibitors^31, 32^, was strongly down-regulated in long bone of Irisin-treated mice^15^. Sclerostin has been defined one of the key proteins involved in mechanical loading, since its expression is decreased by loading^33^ and *SOST* deficient hindlimb unloading mice are resistant to bone loss^34^. Accordingly, here we found that sclerostin expression in long bones of unloaded mice was three-times higher than control mice, whereas its increase was blunted by r-Irisin treatment. At the same time, the expected increase in *Rankl*/*Opg* ratio observed in unloaded mice was maintained at control level by Irisin treatment that specifically attenuated unload-induced reduction in *Opg*. This modulation of Opg expression may be exerted by Irisin as a direct effect on osteoblast precursors or through an indirect osteocyte-mediated action, since it is well known that osteocytes can modulate Rank-l and Opg expression in osteoblasts in order to control osteoclasts formation and activity^35^. Because osteocytes are relatively difficult to isolate and culture *in vitro*, a major limitation of our study is that we cannot ascertain if Irisin directly affects these cells, as we previously demonstrated for osteoblasts, which are a direct target of Irisin *via* a receptor-mediated mechanism^15^. However, since it has been shown that the Rankl/Opg balance is finely tuned by sclerostin^23^, we speculate that, if in unloading conditions Opg and Sclerostin are both maintained at control levels by Irisin, these two effects may be linked together and both contribute to the protection against bone loss.

*In vivo*, r-Irisin treatment decreased OC number in mice kept under normal load^15^. Since it is well-known that bone loss caused by unloading involves increase in OC formation^23–25^, we sought to determine if r-Irisin was effective in inhibiting this effect. Our results revealed that the expected three-fold change increase in OC number, observed in trabecular bone of unloaded mice, was significantly reduced by r-Irisin. In parallel, the unload-induced reduction of new osteoid deposition was significantly increased by r-Irisin, even though it is not restored to the level of normal loading mice. Overall, the combined effect of r-Irisin in stimulating new bone deposition and inhibiting bone resorption, resulted in an unaffected trabecular bone mass of unloaded mice treated with the myokine.

Furthermore, we also investigated whether the muscle atrophy, occurring under mechanical unloading, is prevented by r-Irisin treatment. Accumulating evidence, covering a large number of spaceflight and hind-limb suspension experiments, demonstrated that a rapid loss in muscle weight and myofibrillar protein content occurs during the first 7 days of unloading and that, after this period, there is a much slower and gradual decline of mass and protein content in skeletal muscle^36^. Therefore, we investigated the effect of Irisin on muscle atrophy only in mice subjected to the shorter period of suspension. Our results clearly demonstrated that treatment with r-Irisin totally prevented muscle weight loss and decrease in fiber size. In agreement with the reduction of the mitochondrial content that often accompanies muscle wasting^37^, we observed a decreased mitochondria density and a disorganized mitochondrial network in muscle fibers from unloaded mice in comparison to control mice. In agreement with previous *in vitro* findings^19^, we observed mitochondrial biogenesis in muscle of Irisin-treated mice. Furthermore, we found increased protein levels of COX IV and mRNA levels of *Nrf-1* and *Tfam*, supporting increased functional capability of mitochondria. However, mitochondrial function needs to be further investigated. Accordingly, we observed *in vivo* that Irisin induced increased mitochondrial density and size, as well as a better organized distribution, also confirmed by an increase of VDAC positive muscle fibers^38^. Although we previously showed that treatment with r-Irisin increased the expression of the Irisin precursor, FNDC5, in muscle fibers of young, healthy mice^15^, we did not observe any changes in the number of FNDC5 positive fibers in unloaded mice treated with r-Irisin, either compared to vehicle-treated unloaded mice or to normal mice. In addition, no change in ATP synthase positive fibers was observed between the three groups of mice. However, worth mentioning is the result that co-localization of fibers positive for both FNDC5 and ATP synthase was reduced in unloaded mice, whereas treatment with r-Irisin preserved the same co-localization as in control mice, indicating a possible correlation between the autocrine-induced FNDC5/Irisin expression pattern and the mitochondrial content in skeletal muscle.

The control of the protein balance in skeletal muscle is finely controlled by a series of events regulating new protein synthesis versus protein degradation^39^. It is well-known that skeletal unloading is an atrophic stimulus that leads to a decrease of protein synthesis and an increase of protein degradation. MyHC is the most abundant protein expressed in skeletal muscle and its function is to regulate the contractile process together with actin protein^40^. We showed here that 4 weeks of unloading activity induced a reduction of the expression of both MyHC type I and MyHC type II. However, the loss of MyHC II expression was completely prevented by r-Irisin treatment. We also explored the expression of the different subtypes of myosin type II: MyHC IIα, MyHC IIx and MyHC IIβ, which are classified in this sequence from the slowest to the fastest isoform of myosin type II. Earlier studies on skeletal muscle plasticity have also focused on the different expression of subtypes of MyHC gene family, from which depend the remodelling of the muscle phenotype^40–42^. For instance, it has been showed that high-frequency electrical stimulation can generate a slow-to-fast switch in the direction I → IIα → IIx → IIβ, whereas tonic low-frequency electrical stimulation can induce a fast-to-slow switch in the opposite direction from IIβ → IIx → IIα → to I. However, differences between muscle types may limit the range of possible adaptations and the time of stimulations can also be crucial in fiber-type transitions^41^. Our findings show that, although r-Irisin did not prevent the MyHC I decrease, it increased the expression of MyHC IIx to a level higher than in control mice. The increase of this subtype of MyHC type II might indicate a fast-to-slow switch, as described above. Therefore, this suggests that Irisin, not being able to preserve the loss of content in MyHC type I, could stimulate the transition of fast-type fibers towards the slow phenotype, as countermeasure to mitigate the reduction of slow fibers caused by unloaded-induced muscular atrophy. Although Irisin was found to increase mitochondrial biogenesis and, therefore, high oxidative capacity that primarily occurs in slow muscle fibers, one limitation of our study is that we analysed only the *vastus lateralis*, which is a type of muscle mainly composed by fast twitch fibers^42^. Future studies, designated to analyse the effect of Irisin on different muscle types, could reveal if there are differences in the response to the myokine depending on the type of muscle examined, or even on the timing of unloading. Thus far, several studies highlighted the response of type I fibers to unloading, but less data were reported on type II fibers^43, 44^. Adapting to the disuse of the contractile function seems to be variable in type II fibers, but this variability may be dependent on differences between subjects examined, on different types of muscle analyzed, and different methods and time used to simulate disuse^45^. Most of the studies on hind-limb suspended mice were based on short-term (7–14 days) unloading protocols, in which no alteration of type II fibers was observed. Zhong and colleagues^45^ demonstrated that type II fibers from semimembranosus muscle were also sensitive to the removal of weight bearing, when it was applied for a longer period (21 days).

In our hand, we found that, among subtypes of MyHC II, MyHC IIx is strongly decreased in *vastus lateralis* of mice subjected to 4 weeks of weight bearing removal, whereas MyHC IIβ was not affected. To our knowledge, this is the first evidence reporting that *vastus lateralis*, undergone unloading, was characterized by a ~60% reduction in MyHC IIx expression, and that this decline, not only was completely inhibited by Irisin, but the myokine up-regulated the expression of MyHC IIx to a higher level than in normal mice.

Overall our data reveal for the first time that r-Irisin treatment ameliorates bone loss and muscle atrophy in unloading mice. For long time, there has been controversy regarding Irisin in humans but many of these raised doubts have been dispelled by the recent study of Jedrychowski and colleagues^46^. Our results may support a promising clinical strategy for the prevention and treatment of both osteoporosis and sarcopenia, particularly applicable to those patients who cannot perform physical activity, as occurs during reduced mobility. Moreover, an Irisin-based therapy might also represent a countermeasure for astronauts that, exposed to microgravity during space flight missions, often encounter a severe bone and muscle loss.

## Methods

### Experimental design and animal model

2-months-old C57BL6 male mice (n = 64) were randomly assigned to eight groups: three groups of control mice (2 groups/preventive protocol and 1 group/curative protocol) and five groups of hind-limb suspended mice (2 groups/preventive protocol and 3 groups/curative protocol) which were subjected to the tail suspension procedure, according to recommendations by Wronski and Morey-Holton^47^. The height of the mice hindquarters was adjusted to prevent any contact of the hind limbs with the cage floor, resulting in approximately a 30° head-down tilt. The forelimbs of the animals maintained contact with the cage bottom, allowing the mice full access to the entire cage. Each mouse was singly housed, maintained under standard conditions on a 12/12 hour light/dark cycle and with access to water and regular chow diet ad libitum (Harlan Teklad 2019, SDS, England). Hind-limb suspended mice were treated on the basis of two different protocols. For the preventive protocol, hind-limb suspended mice were treated with vehicle (physiologic water sterilized by 0.22 μ filtration) (n = 8) or with 100 µg kg^−1^ r-Irisin (n = 8) by i.p injection once a week for 4 weeks. r-Irisin was provided by Adipogen International (San Diego, USA). Two groups of control mice (n = 8/group) were also singly housed and one group was treated with vehicle (Rest vehicle-injected mice) and the other group was treated with 100 µg kg^−1^ r-Irisin (Rest Irisin-injected mice) by i.p injection once a week for 4 weeks. For the curative protocol, hind-limb suspended mice were left untreated for 4 weeks and then treated with vehicle (n = 8) or with 100 µg kg^−1^ r-Irisin (n = 8) by i.p injection once a week for the following 4 weeks of suspension. The fifth group consisted of mice (n = 8) that were hind-limb suspended for 4 weeks and then subjected to reload, which involved normal cage activity, and treated with vehicle by i.p injection (Reload vehicle-injected mice) once a week for the following four weeks. Normal weight-bearing mice (n = 8) were treated with vehicle by i.p injection once a week during the last 4 weeks (Rest vehicle-injected mice) of the curative protocol. Mice were weighed once a week and at the end of the experimental procedure were euthanized and their tissues were surgically excised. Right femurs were harvested and employed for µCT analysis. Left femurs and tibia were subjected to bone marrow flushing and then stored in liquid nitrogen for western blot and real-time PCR analysis. The femurs and the tibia were cut longitudinally and the two halves have been mixed and used one pair (half femur and half tibia) for western blot and the other pair for real-time PCR analysis. Lumbar vertebrae (from L1 to L5) were dissected, fixed with 4% (vol/vol) paraformaldehyde for 18 hours at 4 °C and processed for histological analysis. Bilateral vastus lateralis muscles were excised from the quadriceps and processed for further analysis. This animal interventional study is in accordance with the European Law Implementation of Directive 2010/63/EU and all experimental protocols were reviewed and approved by the Veterinary Department of the Italian Ministry of Health (Project 522-2016PR).

### Contact radiography

Long bones were X-rayed by using contact radiography (Vista Scan Combi Durr Dental AG, Bietigheim-Bissingen). Scans were obtained by using a setting of 60 kV, 8 mA and 2.5 sec exposure time. The developed X-rays were scanned into a personal computer for image acquisition. No direct measures were performed on the images.

### Microcomputed tomography analysis of femurs

MicroCT (μCT) scanning was performed to measure morphological indices of metaphyseal regions of femurs. Bone samples were rotated around their long axes and images were acquired using Bruker Skyscan 1172 (Kontich, Belgium) with the following parameters: pixel size = 6 μm3; peak tube potential = 59 kV; X-ray intensity = 167 μA; 0.4° rotation step. Raw images were reconstructed by the SkyScan reconstruction software (NRecon) to 3-dimensional cross-sectional image data sets using a 3-dimensional cone beam algorithm. Structural indices were calculated on reconstructed images using the Skyscan CT Analyzer (CTAn) software (Bruker). Cortical and trabecular bone were separated using a custom processing algorithm in CTAn, based on the different thicknesses of the structures. Cortical bone was analysed by a region of 150 slices, starting 9 mm distal to the metaphysis. Cortical parameters included BMD and cortical thickness (Ct.Th). Trabecular bone was analysed in the proximal metaphysis region starting just distal to the metaphysis and continued distally for 200 slices. Trabecular parameters included BMD, bone volume fraction (BV/TV), number (Tb.N), thickness (Tb.Th), separation (Tb.Sp) and Fractal dimension.

### Histological analysis of vertebrae

Lumbar vertebrae were embedded with MMA after dehydration and the plastic sections were cut by a standard microtome (RM-2155 Leica, Heidelberg, Germany) into 7 μm for von Kossa staining and 5 μm for Tartrate-resistant acid phosphatase (TRAP) and Goldner’s Masson Trichrome staining. The sections were stained by the von Kossa silver impregnation with van Gieson counterstained method to determine cancellous bone volume fraction (BV/TV %), trabecular number (Tb.N, 1/mm) trabecular thickness (Tb.Th, mm) and trabecular separation (Tb.Sp, mm) on L3-L4 vertebrae. For the analysis of osteoclasts (osteoclast number per bone perimeter, OCs/BPm), bone sections were incubated in TRAP staining solution and then counterstained with methyl green. The Goldner’s Masson trichrome stain was performed for the analysis of new osteoid formation. Sections were evaluated under brightfield microscopy after Goldner’s Trichrome staining to determine static parameters of bone surface (BS) and osteoid surface (OS). Histological sections were viewed under a microscope (Leica) using a 40x objective lens and analyzed by using Image-J software^48, 49^.

### Histological analysis of muscle

*Vastus lateralis* muscles were excised from the quadriceps, fixed and embedded in paraffin. 5 µm thick histological sections were cut and stained with hematoxylin and eosin (H&E). All observations were performed with a Nikon Eclipse 80i light microscope (Nikon). The CSA was measured on H&E stained slides at magnification of 20x by using the NIS-Element BR 4.10.00 software.

### Transmission Electron Microscopy (TEM)

*Vastus lateralis* muscle fragments were fixed in 2% glutaraldheyde-2% paraformaldehyde in phosphate buffer (PB) for 4 h at room temperature, post fixed in 1% osmium tetroxide, and embedded in an Epon-Araldite mixture. Semithin sections (2 μm) were stained with toluidine blue. Thin sections were obtained with an MT-X ultratome (RMC; Tucson, AZ), stained with lead citrate, and examined with a CM10 transmission electron microscope (Philips; Eindhoven, The Netherlands).

### Peroxidase Immunohistochemistry

Immunohistochemistry was performed on 3 μm-thick paraffin-embedded sections of vastus lateralis muscle samples. After dewaxing, antigen retrieval was achieved with a pressure cooker treatment (90 °C for 20 min) by soaking sections in a sodium citrate buffer 0.01 M, pH 6.0. After a thorough rinse in phosphate buffered saline (PBS), sections were reacted with 0.3% H_2_O_2_ (in PBS; 30 min) to block endogenous peroxidase, rinsed with PBS and incubated in a 3% normal goat blocking solution (in PBS; 60 min). Then, they were incubated with the polyclonal rabbit anti-VDAC primary antibody (Cell Signaling Technology) overnight at 4 °C. After a thorough rinse in PBS, sections were incubated in a 1:200 v/v biotinylated goat anti-rabbit IgG secondary antibody solution (Vector Laboratories, Burlingame, CA; in PBS; 30 min). Histochemical reactions were performed using Vectastain ABC Kit (Vector Laboratories) and Sigma Fast 3,3′-diaminobenzidine (Sigma-Aldrich) as the substrate. Sections were finally counterstained with hematoxylin, dehydrated and mounted in Entellan. Staining was never observed when the primary antibody was omitted. For morphometric analysis, immunostained tissue sections were observed with a Nikon Eclipse E800 light microscope using a 40x objective, and digital images were captured with a DXM 1200 camera. The percentages of muscle fiber areas occupied by VDAC protein stain (5 high power fields for each section) were determined by the Nikon Lucia IMAGE (v. 4.61) image analysis software. Results are given as mean ± SEM.

### Immunofluorescence

For immunofluorescence, 5 μm thick paraffin sections of vastus lateralis were re-hydrated and antigens were retrieved. Sections were then permeabilized with Triton X-100 0.5% in PBS for 10 minutes at RT and non-specific interactions were blocked with 10% HS in PBS for 30 minutes at RT. After washing in PBS tissues were incubated with primary antibody against FNDC5 (Abcam), ATPsynthase (Invitrogen Molecular Probes), Laminin (Santa Cruz), MyoD (Dako) and Pax7 (R&D Systems). Fluorescent-labeled secondary antibodies were anti-mouse Alexa Fluor®-555 and Alexa Fluor®-594; anti-rabbit Alexa Fluor®-488 and Alexa Fluor®-594; anti-rat Alexa Fluor®-568 (Thermo Fisher Scientific). Nuclei were counterstained with fluorescent mounting medium plus 100 ng/ml 4′,6-diamidino-2-phenylindole (DAPI) (Sigma-Aldrich). At least 5 high power fields were analyzed for each section. Staining was never observed when the primary antibody was omitted. Percentage of positive fibers were calculated by using the NIS-Element BR 4.10.00 software.

### *Ex vivo* primary cell cultures

Bone marrow was flushed from mouse femurs and tibia and cultured in α-MEM (Life Technologies) supplemented with 10% (vol/vol) FBS (Gibco, Life Technologies) and 1% penicillin/streptomycin (Life Technologies). For osteogenic differentiation, bone marrow cells were cultured with α-MEM/10% FBS, supplemented with 50 μg/mL ascorbic acid and 10^−2^ M β-glycerophosphate. At day 10, cells were fixed in 3.7% (vol/vol) formaldehyde for 5 minutes and subjected to alkaline phosphatase (ALP) staining. Image J software was used to calculate area of ALP+ colony forming unit (Cfu-f). For OC differentiation, bone marrow cells were cultured with α-MEM/10% FBS, supplemented with 5 ng/mL of macrophage colony-stimulating factor (MCSF; R&D system) and 3 ng/ml of receptor activator of nuclear factor kappa-B ligand (RANK-L; R&D system). At day 7, cells were fixed in 3.7% (vol/vol) formaldehyde for 5 minutes and stained for TRAP. TRAP+ cells with more than three nuclei were counted as OCs.

### Real Time-PCR

Total RNA from bone and muscle tissues and osteoblast or osteoclast cultures was extracted using spin columns (RNeasy, Qiagen) according to the manufacturer’s instructions and reverse transcription performed using iScript Reverse Transcription Supermix (Bio-Rad). The resulting cDNA (20 ng) was subjected to quantitative PCR (qPCR) using the SsoFast EvaGreen Supermix (Bio-Rad) on an iCycler iQ5 Cromo4 (Bio-Rad). Each transcript was assayed in triplicate and cDNA was normalized to murine Glyceraldehyde 3-phosphate dehydrogenase (GAPDH) and β-Actin. Quantitative measures were obtained using the ΔΔCT method.

### Western Blotting

15 μg of protein from bone and muscle tissues were subjected to 12% SDS-PAGE and subsequently transferred to nitrocellulose membranes (Hybond, Amersham). The blots were probed using primary antibody anti-Sclerostin (Abcam), anti-OxPhos Complex IV subunit I (Invitrogen) and anti-MyHC II (Abcam) and IRDye-labeled secondary antibodies (680/800 CW) (LI-COR Biosciences). For immunodetection, the Odyssey infrared imaging system was utilized (LI-COR Corp., Lincoln, NE). All data were normalized to background and loading controls.

### Statistical analysis

One-way analysis of variance (ANOVA) was used for evaluating the existence of differences among the groups. When significant difference was detected, Bonferroni’s post hoc analysis was used to determine the significance between every two groups. Values of p < 0.05 were considered statistically significant. Cohen’s d values were measured for non-significant differences of results of Figs 1 and 6 and they can be found as Supplementary Table S1.

## Electronic supplementary material

Supplementary Information
