# Supplementary material for: Irisin prevents and restores bone loss and muscle atrophy in hind-limb suspended mice
Source: Sci Rep. 2017 Jun 6;7:2811. doi: 10.1038/s41598-017-02557-8 (PMC5460172; doi:10.1038/s41598-017-02557-8)
Supplement: Supplementary file 1 — Supplementary Information [file 41598_2017_2557_MOESM1_ESM.pdf]

Irisin prevents and restores bone loss and muscle atrophy in hind-limb suspended mice.

[1^]Graziana Colaianni, [1^]Teresa Mongelli, [1]Concetta Cuscito, [1]Paolo Pignataro, [1]Luciana Lippo, [2]Giovanna Spiro, [1]Angela Notarnicola, [3]Ilenia Severi, [4]Giovanni Passeri, [5]Giorgio Mori, [1]Giacomina Brunetti, [1]Biagio Moretti, [6]Umberto Tarantino, [1]Silvia C. Colucci, [7]Janne E. Reseland, [2]Roberto Vettor, [3^]Saverio Cinti, [8^*]Maria Grano.

Supplementary Table S1. Cohen's d values were measured for non-significant differences of results from microCT analysis on Fig. 1 (preventive protocol) and Fig. 6 (curative protocol).

**Bone Parameters Fig. 1 (preventive protocol)**	**Comparison between Groups**	**p value**	**Cohen's d**
Cortical BMD	Unload-Irisin-inj Vs Rest-veh-inj	0.69	0.35
Cortical Th	Unload-Irisin-inj Vs Unload-veh-inj	0.71	0.24
Trabecular BMD	Rest-veh-inj Vs Unload-Irisin-inj	0.10	1.22
Trabecular BMD	Unload-Irisin-inj Vs Unload-veh-inj	0.10	1.51
BV/TV	Rest-veh-inj Vs Unload-Irisin-inj	0.11	1.50
BV/TV	Unload-Irisin-inj Vs Unload-veh-inj	0.12	1.54
Tb.N	Rest-veh-inj Vs Unload-Irisin-inj	0.09	1.38
Tb.N	Unload-Irisin-inj Vs Unload-veh-inj	0.13	1.54
Tb.Th	Unload-veh-inj Vs Rest-veh-inj	0.90	0.07
Tb.Th	Rest-veh-inj Vs Unload-Irisin-inj	0.37	0.80
Tb.Th	Unload-veh-inj Vs Unload-Irisin-inj	0.56	0.52
Tb.Sp	Unload-veh-inj Vs Unload-Irisin-inj	0.23	0.94
Fractal D	Rest-veh-inj Vs Unload-Irisin-inj	0.35	0.64
Fractal D	Unload-Irisin-inj Vs Unload-veh-inj	0.09	1.45

**Bone Parameters Fig. 6 (curative protocol)**	**Comparison between Groups**	**p value**	**Cohen's d**
Cortical BMD	Unload-Irisin-inj Vs Rest-veh-inj	0.19	1.55
Cortical BMD	Reload-veh-inj Vs Unload-Irisin-inj	0.07	2.08
Cortical Th	Unload-veh-inj Vs Unload-Irisin-inj	0.85	0.15
Cortical Th	Reload-veh-inj Vs Rest-veh-inj	0.35	0.72
Trabecular BMD	Rest-veh-inj Vs Unload-Irisin-inj	0.24	0.75
Trabecular BMD	Unload-Irisin-inj Vs Unload-veh-inj	0.45	0.67
Trabecular BMD	Reload-veh-inj Vs Unload-Irisin-inj	0.77	0.24
BV/TV	Rest-veh-inj Vs Unload-Irisin-inj	0.66	0.28
BV/TV	Unload-Irisin-inj Vs Unload-veh-inj	0.08	1.47
BV/TV	Unload-Irisin-inj Vs Reload-veh-inj	0.70	0.30
Tb.N	Rest-veh-inj Vs Unload-Irisin-inj	0.32	0.63
Tb.N	Unload-Irisin-inj Vs Unload-veh-inj	0.31	0.94
Tb.N	Unload-Irisin-inj Vs Reload-veh-inj	0.68	0.46
Tb.Th	Rest-veh-inj Vs Unload-veh-inj	0.72	0.22
Tb.Th	Unload-Irisin-inj Vs Rest-veh-inj	0.70	0.22
Tb.Th	Unload-Irisin-inj Vs Unload-veh-inj	0.60	0.37
Tb.Sp	Unload-Irisin-inj Vs Unload-veh-inj	0.99	0.008
Tb.Sp	Reload-veh-inj Vs Unload-Irisin-inj	0.65	0.30
Fractal D	Rest-veh-inj Vs Unload-Irisin-inj	0.37	0.58
Fractal D	Unload-Irisin-inj Vs Unload-veh-inj	0.23	1.13
Fractal D	Reload-veh-inj Vs Unload-Irisin-inj	0.93	0.09
